# Influence of General Mineral Condition on Collagen-Guided Alveolar Crest Augmentation

**DOI:** 10.3390/ma13163649

**Published:** 2020-08-18

**Authors:** Marcin Kozakiewicz, Piotr Szymor, Tomasz Wach

**Affiliations:** Department of Maxillofacial Surgery, Medical University in Lodz, Pl. Hallera 1, 90-647 Lodz, Poland; marcin.kozakiewicz@umed.lodz.pl (M.K.); piotr.szymor@umed.lodz.pl (P.S.)

**Keywords:** oral surgery, collagen scaffold, intraoral radiograph, image analysis, jaw bone, augmentation, bone substitute material, bone turnover, thyroid-stimulating hormone, densitometry

## Abstract

The local regeneration of bone defects is regulated by general hormone, enzyme, ion, and vitamin levels. General diseases and dysregulation of the human mineral system can impact this process, even in alveolar crest. The aim of this study is to investigate a relation between bone density, measured in two-dimensional X-rays, and general mineral condition of patients. The study included 42 patients on whom tooth extractions were performed. Data were divided into two groups: the region where collagen scaffold (BRM) was used and the reference region of intact normal bone (REF). Two-dimensional intraoral radiographs were taken in all cases just after the surgery (00 M) and 12 months later (12 M). Thyrotropin (TSH), parathormone (PTH), Ca^2+^ in serum, HbA1c, vitamin 25(OH)D_3_, and spine densitometry were checked. Digital texture analysis in MaZda 4.6 software was done. Texture Index (TI: BRM 1.66 ± 0.34 in 00 M, 1.51 ± 0.41 in 12 M, and REF 1.72 ± 0.28) and Bone Index (BI: BRM 0.73 ± 0.17 in 00 M, 0.65 ± 0.22 41 in 12 M, and REF 0.80 ± 0.14) were calculated to evaluate bone regeneration process after 12 months of healing (TI (*p* < 0.05) and BI (*p* < 0.01) are lower in BRM 12 M than in REF). This showed a relation between BI and TSH (R^2^ = 26%, *p* < 0.05), as well as a between BI and patient age (R^2^ = 65%, *p* < 0.001), and a weak relation between TI and TSH level (R^2^ = 10%, *p* < 0.05). This study proved that a collagen scaffold can be successfully used in alveolar crest regeneration, especially in patients with a high normal level of TSH in the middle-aged population.

## 1. Introduction

Systemic conditions affect bone structure [1]. It has been shown that more than 50% of people affected by chronic diseases arbitrarily discontinue therapy within one year [2], intensifying the impact of this known relationship. Takaishi et al. [3] suggested that the possible association between alveolar bone mineral density (measured in intraoral radiographs) and poor general mineral condition was a predictor of vertebral fracture risk. This study was based on the evaluation of optical density. However, the analysis of the optical density histogram does not give much information about bone structure [4], and the correct bone structure provides knowledge about the condition of the bone (i.e., whether it is living and healthy) [5]. In order to extract this information from an intraoral radiograph, a second-order feature is needed. Such features are named texture features and are proposed as objective measures for radiological bone structure monitoring [6]. Based on the analysis of radiological textures, there is a chance of finding compounds of the general mineral condition within the structure of the alveolar bone [7].

Augmentation of the alveolar crest is one of the basic ways to improve bone conditions in the oral cavity before its prosthetic treatment. The regeneration of bone tissue allows clinicians to place implants or miniscrews with greater diameter and length [8], thus allowing better clinical results and durability [9]. The basic biological mechanism used is osteoconduction. It requires scaffolding made of biomaterial on which a new bone is formed, gradually filling the bone defect, e.g., the alveolus. Scaffolding can be built of amorphous calcium phosphate, calcium carbonate, biologically active glass, crystalline calcium phosphate, fluorohydroxyapatite, collagen, or a mixture of collagen and calcium phosphate [10,11,12]. These are usually highly porous granules that are easily spilled in the operating field, but collagen is available in the form of a sponge that can easily be stabilized in the alveolus; hence, there is interest in the collagen-guided augmentation of the alveolar crest.

The aim of this study is evaluation of the influence of selected systemic factors on the formation of bone structure in places of its collagen augmentation within the alveolar crest.

## 2. Materials and Methods

The study was approved by the University Ethical Committee RNN/485/11/KB. Forty-two patients were included in this study (age 50 ± 14 years, 21 females and 21 males, and generally healthy). All 42 patients underwent tooth removal with local anesthesia (4% articaine with 1:100,000 adrenaline, 3 M ESPE AG, Seefeld, Germany).

Inclusion criteria involved two-dimensional radiographs taken immediately after surgery and 12 months later and laboratory tests to check hormone, ion, and vitamin levels: parathormone (PTH, where norm is 10 to 60 pg/mL), thyrotropin (TSH), where norm is 0.23–4.0 µU/mL; calcium in serum (Ca^2+^), where norm is 9–11 mg/dL; glycated hemoglobin (HbA1c), where norm is <5%; and vitamin 25(OH)D_3_ (D3), where norm is 31–50 ng/mL. Spine densitometry, where T-score can be examined, was also considered. T-score shows the ratio between bone mineral density (BMD) of the examined patient to average BMD for young patients. Normal value for normal bone is >−1.0, osteopenia is indicated by values between −1.0 and −2.5, and scores <−2.5 indicate osteoporosis.

Exclusion criteria included a lack of X-rays, defective X-ray images in the visual assessment, lack of laboratory test, complications after surgery, and noncontrolled general diseases. A limitation of the study is that laboratory tests were checked before the surgery and authors assumed that laboratory-tested levels did not change.

Two-dimensional X-ray images were taken immediately after the surgery (00 M) and 12 months after the surgery (12 M). The Digora Optime radiography system was used to take intraoral X-ray images (TYPE DXR-50, SOREDEX, Helsinki, Finland). The radiographs were taken in the standardized way [13] with the following parameters: 7 mA, 70 mV, and 0.1 s (the focus apparatus was from Instrumentarium Dental, Tuusula, Finland). Positioners were used to take images repeatably with a 90° angle of X-ray beam to the surface of phosphor plate. Texture of X-ray images were analyzed in MaZda 4.6 software, developed by University of Technology in Lodz [14], to check how the features changed over the 12 months of observation.

**Figure 1:**
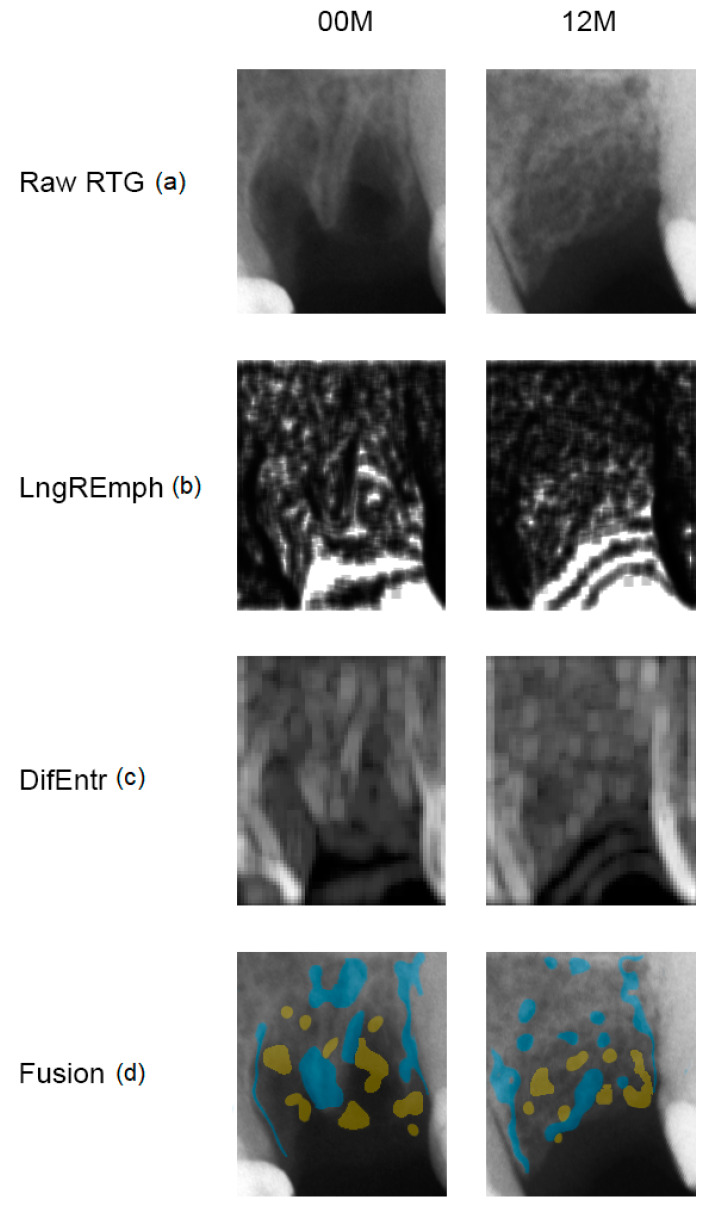
Collagen-guided alveolar crest augmentation. Raw RTG (**a**): intraoral radiograph immediately after collagen implantation into the alveolar (00 M) and at a 12-month follow-up (12 M). LngREmph (**b**): a map of the occurrence of long chains of pixels of similar optical density; brighter fields indicate more of such occurrences in the X-ray image. DifEntr (**c**): map of regions with chaotically arranged small elements recorded in the X-ray; brighter places indicate a more chaotic microstructure. Fusion (**d**): the application of places on the raw X-ray which represent the largest occurrence of long pixel chains (yellow = high LngREmph) in the alveolar crest and the most chaotic places (blue = high DifEntr). Please note that yellow areas do not coincide with blue areas. This is due to the detection of the different structurally different alveolar regions, i.e., LngREmph = radiolucent regions = soft tissue, while DifEntr = trabecular regions = bone.

Data were divided into two groups: the experimental group with collagen scaffold applied 00 M and 12 M (Figure 1), and reference group with intact normal bone. The regions of interest (ROIs) were normalized (μ ± 3σ) to share the same average (μ) and standard deviation (σ) of optical density within the ROI. Selected image texture features (entropy and difference entropy from the co-occurrence matrix and long-run emphasis moment from the run-length matrix) in ROIs were calculated for reference bone and for bone with collagen scaffold applied:

(1) $$Entropy=-\overset{Ng}{\underset{i=1}{\sum }}\overset{Ng}{\underset{j=1}{\sum }}p\left(i,j\right)log(p\left(i,j\right)$$

(2) $$DifEntr=-\sum_{i=1}^{Ng}p_{x-y}\left(i\right)log\left(p_{x-y}\left(i\right)\right)$$

where Σ is sum, *Ng* is the number of levels of optical density in the radiograph, *i* and *j* are optical density of pixels that are 5 pixels distant from one another, *p* is probability, and log is common logarithm [6].

(3) $$LngREmph=\frac{\sum_{i=1}^{Ng}\sum_{k=1}^{Nr}k^{2}p\left(i,k\right)}{\sum_{i=1}^{Ng}\sum_{k=1}^{Nr}p\left(i,k\right)}$$

where Σ is sum, *Nr* is the number of series of pixels with density level *i* and length *k*, *Ng* is the number of levels for image optical density, *Nr* is the number of pixels in series, ad *p* is probability [15,16]. These three equations were subsequently used for the Texture Index construction [12]. Finally the Texture Index (TI) and Bone Index (BI), which represent the ratio of the measure of diversity of the structure observed in the radiograph to the measure of the presence of uniform longitudinal structures, were calculated:

(4) $$Texture\;index=\frac{Entropy}{LngREmph}=\frac{(-\sum_{i=1}^{Ng}\sum_{j=1}^{Ng}p\left(i,j\right)log\left(p\left(i,j\right)\right))\sum_{i=1}^{Ng}\sum_{k=1}^{Nr}p\left(i,k\right)}{\sum_{i=1}^{Ng}\sum_{k=1}^{Nr}k^{2}p\left(i,k\right)}$$

(5) $$Bone\;Index=\frac{DifEntr}{LngREmph}=\frac{(-\sum_{i=1}^{Ng}p_{x-y}\left(i\right)log\left(p_{x-y}\left(i\right)\right))\sum_{i=1}^{Ng}\sum_{k=1}^{Nr}p\left(i,k\right)}{\sum_{i=1}^{Ng}\sum_{k=1}^{Nr}k^{2}p\left(i,k\right)}$$

The index defined in this way (Equation (5)) was taken as a measure of bone regeneration. The Shapiro–Wilk test was used for normality testing. The average values of the texture features were compared by Student’s *t*-test when normal distribution was confirmed, or Mann–Whitney (Wilcoxon) W-test was used to compare medians when non-normal distribution was found. Comparisons between different general mineral condition parameters were performed with the one-way ANOVA or the Kruskal–Wallis test depending on the presence of normal distribution. Simple regression analysis was also done for the investigation of relationships between general mineral condition parameters and radiological texture features. When *p* < 0.05, it was assumed that the difference was statistically significant. Stargraphics Centurion 18 ver.18.1.12 (StarPoint Technologies, Inc., Addison, TX, USA) was used for statistical analyses.

## 3. Results

Since the *p*-value is greater than or equal to 0.05, there is not a statistically significant difference between the medians at the 95% confidence level in Mann–Whitney (Wilcoxon) W-test for TI immediately after collagen implantation (W = 941, *p* = 0.601); this was also the case for BI (W = 1094, *p* = 0.058). On the contrary, after 12 months, a significantly lower value was found for both indices compared to the reference bone: for TI, *t* = −2.189 and *p* < 0.033; for BI, *t* = −3.041 and *p* = 0.004 (Table 1). The variables describing BI are shown in Table 2.

**Figure 2:**
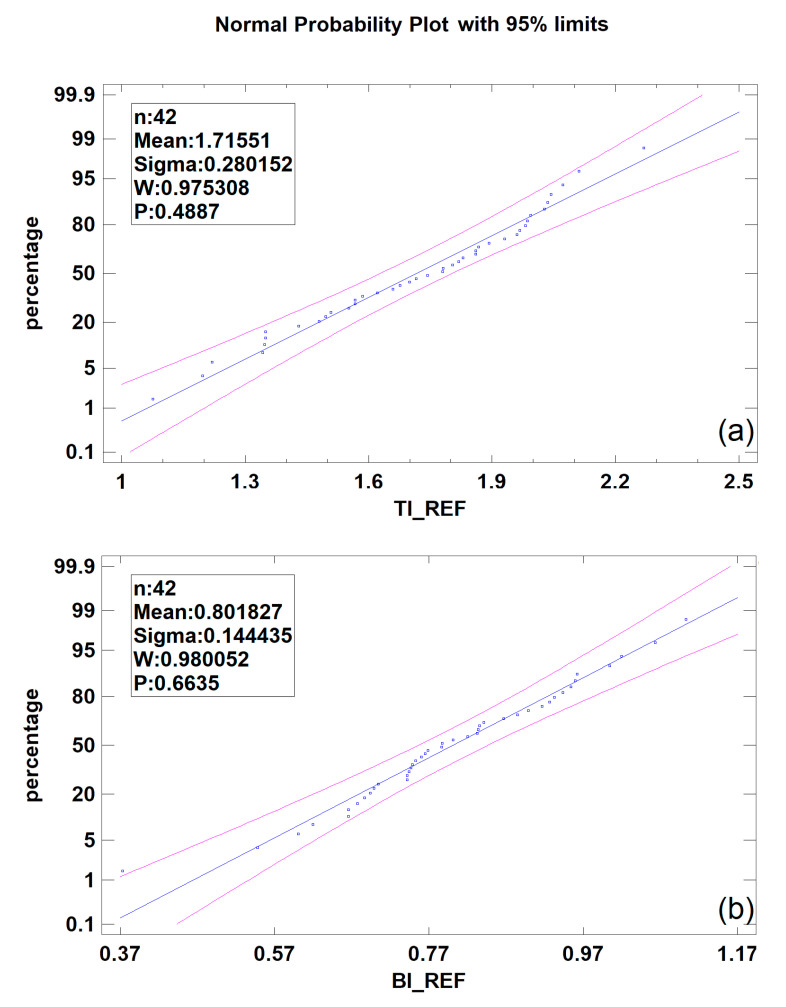
Distribution of Texture Index (TI)—(**a**) and Bone Index (BI)—(**b**). Note: n, number of cases; W, Shapiro–Wilk test result; P, statistical significance.

TI calculated for reference bone is not dependent on age, T-score, PTH, vitamin D3, or Ca, but a relatively weak relationship to TSH serum level was noted (linear regression model *p* < 0.05, CC = −0.32, R^2^ = 10%) for the reference bone in alveolar crest. In contrast, BI is completely independent of the biochemical parameters tested. Both indices have a normal distribution in the given population, i.e., TI: Shapiro–Wilk test 0.98, *p* = 0.48; BI: Shapiro–Wilk test 0.98, *p* = 0.66 (Figure 2).

**Figure 3:**
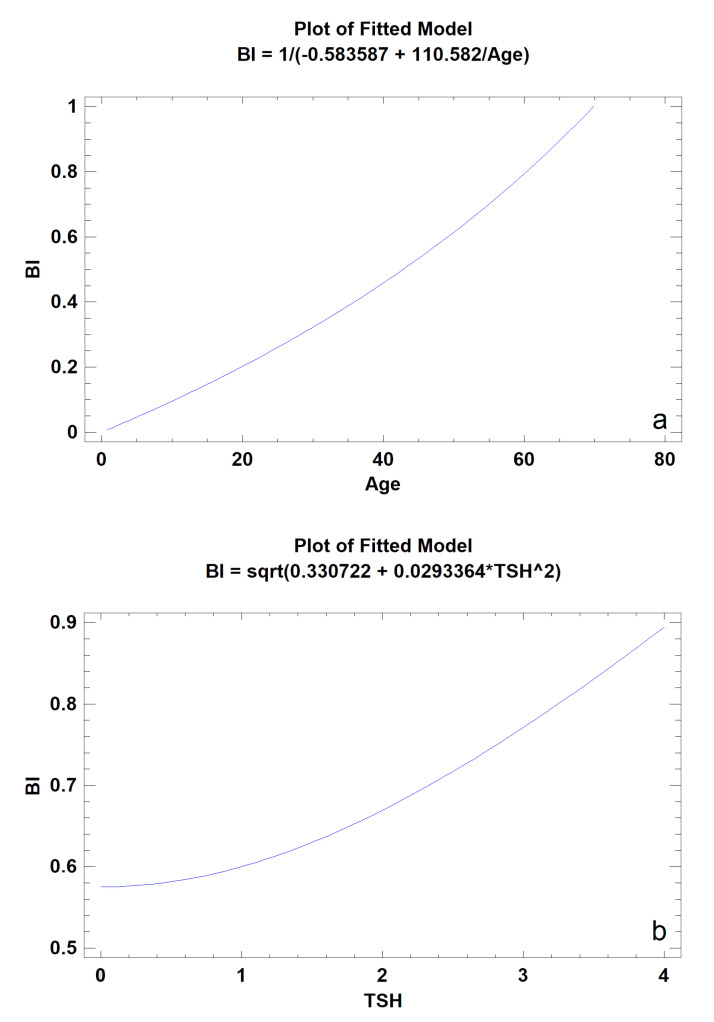
The models of simple regression describing the relationship of the 12-month bone regeneration outcome, expressed as BI calculated from intraoral radiographs, to patient′s age (**a**) and TSH level (**b**) (*p* < 0.05).

A moderately strong relationship was found between BI and the patient’s age (*p* < 0.001); a similar relationship was also found between BI and TSH serum level (*p* < 0.05) (Table 3). Older patients had higher BI values (BI = 1/(−0.583587 + 110.582/Age)). Similarly (Figure 3), in patients with higher serum TSH concentrations (but remaining in the physiological norm), higher values of this index were observed (BI = sqrt(0.330722 + 0.0293364∗TSH^2)). Neither BI (*p* = 0.688) nor TI (*p* = 0.961) were statistically related to osteopenia defined on the basis of T-score (<−1.0) in the tested group of patients.

## 4. Discussion

The assessment of the distribution of sites with high LngREmph and high DifEntr values leads to interesting findings. Firstly, these sites never overlap (Figure 1). Secondly, the LngREmph mainly indicates those places in the intraoral radiograph where trabeculae in the alveolar bone are not visible. Contrarily, DifEntr indicates the sites where a dense network of trabeculae is located. Thus, entropy measuring is a good bone tissue marker [6], contrary to LngREmph which is a good marker of bone atrophy. Thirdly, the known Texture Index [12], defined as the ratio of the entropy of a bone image to the long run-length emphasis moment, is a good indicator to be used in the search for newly regenerated bone or the location of reflexive bone in the alveolar process. The above observations lead to an attempt to calculate a modified texture index as a ratio of DifEntr to LngREmph. This modified texture index is called the Bone Index (BI). Both indices do not indicate that collagen implantation sites are different from the reference bone on the day of surgery. However, BI is on the threshold of significance (*p* = 0.058). This can be seen as a positive feature of BI sensitivity because it is clear that there is not yet normal bone at the site after surgery. The lack of sensitivity of both indices in this range can be explained by the total translucency of the implanted collagen to the radiation creating the X-ray image and the existence of walls of normal bone overlapping the image of the surgical site in the intraoral image.

The result of crestal bone healing in a 12-month observation monitored by BI indicates that the regenerated bone is not identical to the reference one (Table 1). Nature creates bones differently than implanted collagen scaffold can (*p* < 0.01). Numerous fields without dense trabeculation after 12 months of healing can be observed (Figure 1). This may be related to the fact that the bone regenerates under stress relief conditions. However, bones loaded, e.g., by dental implants [17], are rebuilt towards compact bone. Decreasing of texture entropy in surrounding bone was observed [7].

Usually, most of the augmentation site is filled with biomaterial and not bone after several postoperational months [18]. This is especially true for hydroxyapatites [19], but it is also observed, although to a lesser extent, in tricalcium phosphates [20,21]. Therefore, the use of materials with collagen scaffolding and filled with tricalcium phosphate has greater potential than collagen alone. Such a blended material is much more similar (taking into account texture analysis) to reference bone than other substitution materials. The collagen–mineral blend implantation has a similar effect to that of collagen material (Osteovit). Contrary to collagen scaffold for bone regeneration, the augmentation result with classical granular biomaterials (tricalcium phosphates) is such that the difference cannot be detected. The reduced value of Texture Index observed in absolute values during one year, indicates the need for further observation of the effectiveness of collagen alone, as well as that of inorganic materials suspended in collagen base, despite both of them being excellent in handling bone defects [12].

The bone structure at the site of collagen implantation, as expressed by the Bone Index, is not related to the measured bone turnover parameters. However, it is related to the patient’s metabolism (TSH serum level), which may indicate that bone regeneration in a short period of 12 months is not strongly influenced by the general mineral condition of a generally healthy patient (in the study, most patients were normal in terms of T-score, 11 had osteopenia, and only 2 had osteoporosis). The basic mechanisms remain unknown because TSH did not affect the proliferation or differentiation of primary osteoblasts, nor did it activate the cAMP (cyclic adenosine monophosphate), p38 mitogen-activated protein kinase, or protein kinase B signal pathways [22]. TSH in vitro inhibited osteoblastogenesis and decreased expression of type I collagen, bone sialoprotein, and osteocalcin [23]. Sampath et al. [24] and Baliram et al. [25] showed that TSH stimulates osteoblast differentiation and function. In humans, TSH also stimulated proliferation and differentiation, as measured by alkaline phosphatase, and increased expression of IGF-1 (Insulin-like Growth Factor 1) and IGF-2 (Insulin-like Growth Factor 2) mRNA, along with complex regulatory action on IGF-binding proteins (insulin-like growth factor-binding protein) and their protease s [26]. Overall, the findings have been interpreted as suggesting that changes in TNFα (Tumor Necrosis Factor α), RANKL (Receptor Activator for Nuclear Factor Ligand), OPG (Osteoprotegerin), and IL-1 (Interleukin 1) signaling in response to TSH may be mediated by the alternative G-protein rather than by cAMP [23,27,28]. Thus, although the TSH receptor is expressed in osteoblasts building the alveolar crest, data from in vitro studies are contradictory, suggesting that TSH may inhibit, amplify, or have no effect on the differentiation of osteoblasts and their function. Moreover, the physiological pathway of the second messenger, which lies below the active TSH receptor in the osteoblasts [29], has not yet been determined. Unfortunately, a different signaling pathway is the key one regulating craniofacial patterning. The bone morphogenic protein (BMP) pathway regulates development of mineralized structures such as the jawbone [30]. Thus, BMP signaling contributes both to shape and functionality of our facial features [31]. BMP signaling also regulates jawbone remodeling and healing [32]. BMP function will contribute to our ability to rationally manipulate this signaling network in the context of tissue engineering. It seems impossible for the TSH and BMP signaling pathways to be intertwined somewhere in the jaw area.

Patients with higher levels of TSH (untreated hypothyroidism) can reveal worse bone regeneration healing results. This may be due to a slowed bone formation process and bone resorption process [33]. This can be considered as confirmation of the results in our study, where good results in the bone regeneration process are related with higher levels of TSH but are still in the norm.

The first link between osteoporosis and bone loss in the oral cavity was discovered sixty years ago [34]. This association is still a contentious issue because of mixed results in the literature, and studies have yet to show conclusively an association between osteoporosis and changes to the residual alveolar ridge [35,36]. When compared with other risk factors (age, body size, bone turnover markers, and T-score), alveolar bone mineral density measurements showed a higher association with vertebral and long bone fractures than lumbar bone mineral density and could successfully identify those patients who had sustained a fracture in the multivariate analysis. Assessment of alveolar bone density [3], contrary to alveolar textural features, may be a useful adjunct method for assessing patients of advanced age during routine dental examinations to monitor the clinical picture and the potential course of osteoporosis. It seems this does not apply to younger patients, i.e., the ones studied in this experiment.

It is worth noting that the Bone Index has a very strong correlation to the age of the patient (correlation coefficient = 81, *p* < 0.001). This suggests the influence of general health on the structure of the regenerated bone. This study cannot clarify this relationship because of the relatively small group of examined patients, short observation time, and limited panel of performed biochemical tests. The potential reasons in tested patients may include genetic factors [37,38], poor dietary intake [39,40], lower physical activity [41,42,43], or decreased level of immune host response in older patients. Jabłońska, in her study, showed that the strain placed on the immune system over time leads to inefficiency that increases with age, with cellular and humoral immunity decreasing as one gets older. Thus, it can only be assumed that low host immune response to substitution material proteins (i.e., collagen) allows for good regeneration of bone defects versus an adverse immunological reaction against xenogenic proteins, but this requires a larger group of observed patients to determine [44]. Another explanation for the difficulty in discovering the influence of general mineral condition on the structure of jaw bones is the specificity of the dental system, i.e., overwhelming local factors. It is worth recalling that within the oral cavity, the skeleton is closer to the external world than all other body locations. The gingiva covers the bone with a layer as thin as 1 mm. Nowhere else is the skeleton this close to the external environment. The presence of teeth growing out of the skeleton and protruding into the external environment, the pressure of multiple chewed foods, the antigenic effect of foods, and the bacterial flora of the oral cavity must significantly affect the structure of the deeper lying bone as well as the process of controlled bone regeneration.

The limitations of this study are a small sample, two-dimensional texture analysis, and a limited panel of biochemical tests. Extrinsic collagen (xenogenic) may cause an adverse reaction, which would not be without an effect on bone regeneration.

## 5. Conclusions

Within the limitations of the present report, it has been demonstrated that the bone formed in the site of regeneration based on a collagen scaffold does not reproduce bone with identical structure to bone formed during ontogenesis. However, as with other bone substitute materials, the collagen scaffold can be used for further dental treatment procedures. The effect of general mineral condition is substantially modified by local factors specific to the alveolar process of the jaw, although its strongest indirect representatives are responsible for a significant effect (TSH and age).

## Figures and Tables

**Table 1:** Calculated Bone Index (BI) and Texture Index (TI) in intraoral radiographs.

Index	00 M	12 M	REF	Difference to Reference
BI	0.73 ± 0.17	0.65 ± 0.22	0.80 ± 0.14	*p* < 0.01 for 12 M
TI	1.66 ± 0.34	1.51 ± 0.41	1.72 ± 0.28	*p* < 0.05 for 12 M

Abbreviations: 00 M, collagen implantation site immediately after surgery; 12 M, collagen implantation site 12 months after surgery; REF, reference trabecular bone.

**Table 2:** Investigated parameters of the general mineral condition.

Summary Statistic	T-Score	PTH	D3	Ca	TSH	D3·Ca/PTH	D3/PTH	lgSQRT Ca/PTH
Average ± Stnd.deviation	−0.36 ± 1.33	38.95 ± 16.77	23.39 ± 10.89	9.71 ± 0.40	1.86 ± 0.91	6.70 ± 3.91	0.70 ± 0.42	−0.28 ± 0.10
Median	−0.2	37.25	20.92	9.61	1.81	4.90	0.51	−0.30
Minimum	−2.9	11.7	10.0	8.8	0,4	2.3	0.2	−0.5
Maximum	2.9	96.3	54.0	10.7	5.05	15.5	1.66	−0.02
Range	5.8	84.6	44.0	1.9	4.66	13.2	1.42	0.47
Stnd. skewness	0.36	3.09 *	2.36 *	1.33	3.32 *	1.55	1.65	1.75
Stnd. kurtosis	−0.12	3.41 *	1.60	−0.05	3.64 *	−0.61	−0.49	1.18

Abbreviations: PTH, parathormone; D3, vitamin 25(OH)D_3_; Ca, calcium concentration in serum; TSH, thyroid-stimulating hormone; lg, common logarithm (used for normalization); SQRT, square root (used for normalization); *, lack of normal distribution.

**Table 3:** The relationship of the 12-month bone regeneration outcome, expressed as BI, to general mineral condition.

Parameter	CC	R^2^	*p*
Age	0.81	65%	0.0003 *
T-Score	0.37	14%	0.260
PTH	0.05	0.3%	0.852
D3	0.52	27%	0.234
Ca	−0.43	18%	0.111
TSH	0.51	26%	0.049 *
D3·Ca/PTH	−0.36	13%	0.428
D3/PTH	−0.34	12%	0.450
lgSQRT_Ca/PTH	−0.29	8%	0.294

Abbreviations: CC, correlation coefficient; R^2^, percentage of data explained by the model; *p*, statistical significance level; PTH, parathormone; D3, vitamin 25(OH)D_3_; Ca, calcium concentration in serum; TSH, thyroid-stimulating hormone; lg, common logarithm (used for normalization); SQRT, square root (used for normalization); *, statistical significance.
